# Assessment of Whole Genome Amplification for Sequence Capture and Massively Parallel Sequencing

**DOI:** 10.1371/journal.pone.0084785

**Published:** 2014-01-07

**Authors:** Johanna Hasmats, Henrik Gréen, Cedric Orear, Pierre Validire, Mikael Huss, Max Käller, Joakim Lundeberg

**Affiliations:** 1 Science for Life Laboratory, School of Biotechnology, Division of Gene Technology, Royal Institute of Technology, Stockholm, Sweden; 2 Department of Forensic Genetics and Forensic Toxicology, National Board of Forensic Medicine, Linköping, Sweden; 3 Clinical Pharmacology, Department of Health and Medical Sciences, Linköping University, Linköping, Sweden; 4 Genomics Unit, Institut Gustave Roussy, Villejuif, France; 5 Department of Pathology, Institut Mutualiste Montsouris, Paris, France; 6 Science for Life Laboratory, Department of Biochemistry and Biophysics, Stockholm University, Stockholm, Sweden; Ohio State University Medical Center, United States of America

## Abstract

Exome sequence capture and massively parallel sequencing can be combined to achieve inexpensive and rapid global analyses of the functional sections of the genome. The difficulties of working with relatively small quantities of genetic material, as may be necessary when sharing tumor biopsies between collaborators for instance, can be overcome using whole genome amplification. However, the potential drawbacks of using a whole genome amplification technology based on random primers in combination with sequence capture followed by massively parallel sequencing have not yet been examined in detail, especially in the context of mutation discovery in tumor material. In this work, we compare mutations detected in sequence data for unamplified DNA, whole genome amplified DNA, and RNA originating from the same tumor tissue samples from 16 patients diagnosed with non-small cell lung cancer. The results obtained provide a comprehensive overview of the merits of these techniques for mutation analysis. We evaluated the identified genetic variants, and found that most (74%) of them were observed in both the amplified and the unamplified sequence data. Eighty-nine percent of the variations found by WGA were shared with unamplified DNA. We demonstrate a strategy for avoiding allelic bias by including RNA-sequencing information.

## Introduction

The introduction of massively parallel DNA sequencing has massively increased the amount of genetic information that can be generated from tissue and cell samples [1]. Genome-wide analyses of genetic structure are particularly valuable in cancer research, where they can provide important information on the origins of the disease and the optimal course of treatment. However, the quantity of tissue available for study is often limited. Therefore, to facilitate detailed analyses of tumor heterogeneity, there is a need for highly sensitive methods that can efficiently amplify the genomes of cancer cells from small samples and for sequencing the functional parts of the genome. This is not only true for cancer research, metagenomic studies of environmental viruses and microbial communities also deal with low-copy number and heterogeneous DNA composition where the biases of amplification techniques also are of importance [2], [3], [4], [5]. Whole genome amplification (WGA) [6], [7], [8] and target enrichment [9], [10], [11] are valuable techniques that are finding increasingly common usage in established cancer research pipelines [12], [13], [14]. Numerous reagents and commercial sequence capture kits have been developed for these purposes, and comparative reviews indicate that most of them are very effective for targeted exome capture [15], [16], [17]. A recent study used WGA in conjunction with exome sequence capture to analyze genomic variation in kidney cancer cells at the single nucleotide level [18], [19]. The results obtained demonstrated that the combination of these two methods provides a powerful tool for identifying new disease-causing mutations even when working with very small quantities of input genetic material. Multiple displacement amplification is suitable for mutation analysis because it has both high resolution and genome coverage and also high accuracy at the nucleotide level, making it superior to degenerate oligonucleotide primed amplification for identifying novel causative mutations. However, there is a need to fully investigate the limitations and scope of this combined approach. Still there areas that have not been explored and the use of an amplification method before target enrichment might induce more false positives, introduce a bias due to low copy number as starting material and therefore comparison of exome sequencing of unamplified and whole genome amplified material are warranted.

A number of approaches can be used to evaluate the bias introduced when using these two technologies together and to validate identified genetic variations. These include performing analyses on unamplified material (although the availability of a sufficiently large sample may prove limiting here), PCR cloning and Sanger sequencing of genome regions, performing replicate runs (possibly using alternate reagents), and the use of complementary RNA sequencing, among others. The latter two methods are probably most suitable for validation on a global scale, and RNA sequencing has the added advantage that it can be used to confirm the expression of mutated alleles. However, it should be noted that Sanger sequencing of amplicons remains the gold standard in mutation analysis.

This paper describes an investigation into the performance of WGA using the phi29 polymerase followed by exome sequence capture and massively parallel sequencing of lung cancer tumor material. To assess the biases of this approach, we sequenced unamplified material and also performed RNA sequencing. Based on our findings, we propose a strategy for identifying biologically relevant variations.

## Materials and Methods

### Samples

Samples from sixteen patients diagnosed with non-small cell lung cancer (NSCLC) were obtained from the Institut de Gustave Roussy and Institut Mutualiste Montsouris (IMM), Paris. All participants gave their written informed consent to participate in the study. Both tumor tissue samples and healthy samples were obtained from each patient and extracted to isolate their genomic DNA and RNA. Microscope analysis indicated that the tumor cell content of the tumor samples was >70%. Genomic DNA was isolated from peritumoral tissue using the QIAamp DNA Mini Kit (Qiagen, Hilden, Germany), which produced eluates with DNA concentrations ranging from 2 to 41.6 ng/ul. RNA samples were extracted according to the manufacturer’s instructions using the Trizol Reagent from Invitrogen. The obtained RNA concentrations ranged from 102.2 to 2060.4 ng/ul. The ethical guidelines of the CHEMORES consortium (www.chemores.org) were observed during this work, which was conducted with the aim of generating a systems biology database for studying resistance to chemotherapy. The study was approved by the local ethics committee, the Institutional Ethics Committee of the IMM RECHERCHE, Institut Mutualiste Montsouris, Paris, France.

### Whole Genome Amplification (WGA)

DNA amplification was performed using the Illustra GenomiPhi V2 DNA Amplification kit (GE Healthcare, Waukesha, Wisconsin) with random hexameric primers, according to the manufacturer’s instructions [14], [15]. The samples were subjected to gel electrophoresis to confirm that the WGA reaction has been successful (data not shown).

### Target Enrichment of WGA Material

Following WGA of the DNA from the tumor cells and the healthy samples, sequence capture was performed following the protocol provided with the solution-based Roche Nimblegen EZ kit (Roche-NimbleGen, Madison, WI, USA). Concentrations were measured using Qubit, after which libraries were prepared for Illumina sequencing. The samples were multiplexed and clustered on a cBot cluster generation system, using a paired-end read cluster generation kit according to the manufacturer’s instructions. The samples were spiked with 1% PhiX Library for quality control purposes and sequenced on an Illumina HiSeq2000 instrument using paired end 2×100 bp technology (Illumina, San Diego, CA, USA). Base conversion was achieved using Illumina’s OLB v1.9 software.

### Target Enrichment of Unamplified Material

For comparative purposes, sequence capture of unamplified tumor DNA material was achieved according to the protocol provided with the SureSelect Human All Exon V4 kit (Agilent, Santa Clara, CA, USA). Library preparation and sequencing were performed using the HiSeq2000 instrument as described above.

### Alignment and Variant Calling of Genomic DNA

The reads obtained by sequencing the DNA samples were quality checked using FastQC, and aligned against the reference genome HG19 using Mosaik-aligner version 2.1 (http://code.google.com/p/mosaik-aligner/). The coverage and total number of reads were evaluated for each sample. Variant calling was performed using GATK version 1.5.2.1 and was based on positions having >10-fold coverage with a phred scaled quality score above 30 in order to reduce the number of false positives. The total numbers of heterozygous and homozygous variants as well as the ratio of the two were calculated for each sample. The ratio of transversions to transitions was calculated for each sample using custom scripts.

### Comparison of WGA and Unamplified Sequencing of Tumor DNA

To evaluate the reliability of WGA as a sample preparation method for exome capture, we compared single nucleotide variant (SNV) calls obtained using WGA to those obtained using unamplified sequencing. First, the genetic variants were filtered to include only exonic regions (CCDS) covered by both the Nimblegen Sequence Capture and Agilent SureSelect. The numbers of genetic variants found by WGA, unamplified sequencing, or both were then extracted using custom scripts and compared using Venn diagrams for each patient. The coverage of genetic variants found uniquely by WGA and unamplified sequencing was also investigated, along with the coverage of shared genetic variants. In addition, we examined the ratio of variant reads to total reads for sequencing following WGA and unamplified sequencing.

To evaluate the effect of coverage filtering on the observed discrepancies between genetic variants identified using WGA and without amplification, the alignment files for the unamplified sequences (BAM files without coverage filtering) were inspected to check for the presence of variants identified only by WGA and vice versa.

### Identification of Tumor Specific Mutations

Tumor specific mutations were identified by removing variants found by sequencing after WGA of the patient’s normal tissue DNA from the variants found by sequencing of WGA tumor tissue DNA. The total number of exonic mutations for each patient was calculated. To evaluate the performance of sequencing following WGA in identifying mutations, the mutations found in each patient were compared to genetic variants found by sequencing unamplified tumor tissue.

The ratio of tumor specific mutations to the total number of genetic variants was also investigated for variants found exclusively by sequencing following WGA as well as for those identified by unamplified sequencing alone and those identified by both methods (shared variants).

### RNA Sequencing, Alignment and Variant Calling

Eleven of the 16 tumor tissue samples provided RNA of sufficient quality and quantity for analysis. These samples were sequenced using the RNA TruSeq kit (Illumina) and a Illumina HiSeq2000 instrument, and aligned to the reference genome (HG19) using Tophat (version 1.0.14). Cufflinks (version 0.8.3) was used to compute FPKM values, and variants having >10-fold coverage and quality scores above 30 were called by mpileup (Samtools). Samtools was used since GATK gave inconclusive variant calls in this pipeline.

To further evaluate the performance of WGA in mutation identification, we analyzed the BAM files obtained during RNA sequencing to determine whether they reflected the genotype calls obtained by sequencing WGA DNA.

To evaluate the use of RNA sequencing for variant calling, we investigated the number and fraction of bi-allelically expressed genes for all variants found by sequencing both WGA and unamplified material. To ensure that only bi-allelically expressed genes were considered, genes were excluded from the comparison unless they had at least one heterozygous variant according to RNA sequencing and were in the top 75% of genes with an FPKM value of at least one.

## Results

### Patients

The patients’ characteristics are presented in Table 1. All patient samples were analyzed by sequence capture and massively parallel sequencing either after WGA or using unamplified material. In addition, RNA sequencing was performed on a subset of samples. Exome sequencing following WGA yielded an average of 36 M reads/patient with a sequence quality of at least 20 and 60X on target coverage for tumor samples, and an average of 34 M reads/patient with a sequence quality of at least 20 and 67X on target coverage for normal samples. Exome sequencing of unamplified tumor material generated a total of 39 M reads/patient with a sequence quality of at least 20 and 74X on target coverage (see Table 2).

**Table 1:** Patient characteristics.

Patient	Gender	Chemo	Relapse	pT	pN	pM	Cancersubtype	Smoking status	UnamplifiedDNA (Tumor)	WGA DNA(Normal/Tumor)	RNA (Tumor)
**118**	Female	–	Yes	2	0	0	AC	Current	✓	✓	✓
**127**	Female	–	No	2	0	0	AC	Current	✓	✓	
**140**	Male	–	No	2	0	1	AC	Former	✓	✓	✓
**146**	Male	Cisplatin/Vinorelbine	Yes	4	1	0	AC	Former	✓	✓	
**210**	Female	Cisplatin/Vinorelbine	Yes	1	2	0	AC	Former	✓	✓	✓
**225**	Female	Cisplatin/Vinorelbine	No	2	2	0	AC	Current	✓	✓	✓
**247**	Female	Cisplatin/Vinorelbine	Yes	N/A	N/A	0	AC	Current	✓	✓	
**255**	Male	–	Yes	2	2	0	AC	Former	✓	✓	✓
**278**	Male	Cisplatin/Vinorelbine	No	2	2	0	AC	Current	✓	✓	✓
**295**	Female	–	Yes	2	0	0	AC	Former	✓	✓	✓
**322**	Male	Cisplatin/Vinorelbine	Yes	2	2	0	AC	Former	✓	✓	
**344**	Male	Cisplatin/Vinorelbine	No	3	1	0	AC	Former	✓	✓	✓
**396**	Male	–	Yes	2	0	0	AC	Current	✓	✓	✓
**412**	Male	–	No	2	1	0	SCC	Current	✓	✓	✓
**421**	Female	Cisplatin/Vinorelbine	Yes	1	2	0	AC	Current	✓	✓	✓
**541**	Female	–	No	1	1	0	AC	Current	✓	✓	

**AC = **Adenocarcinoma.

**SCC = **Squamous cell carcinoma.

**pT = **Postsurgical histopathological classification of primary tumour.

**pN = **Postsurgical histopathological classification of regional node.

**pM = **Postsurgical histopathological classification of distant metastasis.

**Table 2:** Summary of variant calling following DNA sequencing.

WGA tumor	TotalHetero	TotalHomo	RatioHetero/homo	Transition/Transversion rate	Coverage intarget regions	Totalreads
118(T)	7359	4136	1.78	3.42	39.67	35.55 M
127(T)	8068	4627	1.74	3.32	72.28	37.43 M
140(T)	7904	4411	1.79	3.25	59.33	34.73 M
146(T)	8104	4247	1.91	3.23	61.05	34.47 M
210(T)	7806	4333	1.80	2.96	53.72	54.23 M
225(T)	8088	4258	1.90	2.91	83.70	43.54 M
247(T)	7091	3498	2.03	2.58	24.65	19.77 M
255(T)	7387	4215	1.75	3.29	62.69	33.76 M
278(T)	8328	4612	1.81	3.11	107.10	57.96 M
295(T)	7517	4451	1.69	3.24	48.06	28.13 M
322(T)	9019	4173	2.16	2.53	49.57	19.14 M
344(T)	8107	4300	1.89	3.08	59.31	29.42 M
396(T)	7408	4169	1.78	3.06	31.25	35.00 M
412(T)	8755	4752	1.84	2.84	98.08	45.19 M
421(T)	8552	4489	1.91	2.95	81.62	37.04 M
541(T)	9669	4511	2.14	3.11	35.20	37.75 M
**Average**	8073	4324	1.87	3.06	60.46	36.44 M
**StDev**	683	286	0.14	0.25	23.45	10.40
**Unamplified tumor**						
118(T)	9006	4823	1.87	2.95	48.39	26.08 M
127(T)	14029	3152	4.45	3.27	87.78	32.40 M
140(T)	8725	4875	1.79	3.16	64.98	35.76 M
146(T)	15193	3123	4.86	3.18	85.07	29.08 M
210(T)	8860	5117	1.73	3.17	79.28	26.47 M
225(T)	14270	3081	4.63	3.30	73.46	24.50 M
247(T)	8879	5066	1.75	3.22	108.08	53.61 M
255(T)	8743	4891	1.79	3.26	81.93	42.86 M
295(T)	8717	5067	1.72	3.03	54.27	110.79 M
278(T)	8656	4998	1.73	3.21	88.89	47.00 M
322(T)	8650	4991	1.73	3.11	73.08	37.62 M
344(T)	14161	3006	4.71	3.15	77.05	26.14 M
396(T)	7627	4455	1.71	3.27	36.48	17.41 M
412(T)	8473	4940	1.72	3.21	73.48	38.37 M
421(T)	9085	4977	1.83	3.14	81.82	45.31 M
541(T)	10900	5329	2.05	3.17	73.85	37.67 M
**Average**	10248	4493	2.50	3.18	74.24	39.44 M
**StDev**	2574	855	1.29	0.09	17.11	21.30
**WGA normal**						
118(N)	7476	4154	1.80	3.43	73.07	38.26 M
127(N)	9290	4619	2.01	2.64	101.20	23.63 M
140(N)	7288	3984	1.83	3.41	59.76	35.17 M
146(N)	8292	4613	1.80	3.32	67.14	37.40 M
210(N)	7894	4334	1.82	3.13	48.37	39.87 M
225(N)	11154	4935	2.26	2.07	127.39	75.50 M
247(N)	8925	4940	1.81	3.27	123.26	37.12 M
255(N)	8560	4771	1.79	3.25	83.78	26.35 M
295(N)	6506	3414	1.91	3.18	27.59	29.18 M
278(N)	6961	3966	1.76	3.10	30.67	28.21 M
322(N)	7747	4610	1.68	3.29	56.95	25.81 M
344(N)	8082	4298	1.88	3.09	60.13	17.95 M
396(N)	7276	4035	1.80	3.18	27.70	34.08 M
412(N)	8010	4156	1.93	2.84	38.52	26.65 M
421(N)	8561	4579	1.87	3.11	64.07	30.20 M
541(N)	10411	4840	2.15	3.03	78.53	46.44 M
**Average**	8277	4391	1.88	3.08	66.76	34.49 M
**StDev**	1224	423	0.15	0.34	30.79	13.08

### Variant Calling after DNA Sequencing

Genetic variants in all samples were called using GATK [20], giving heterozygote:homozygote ratios ranging from 1.7 to 2.3 in samples subjected to WGA, and from 1.7 to 4.9 for unamplified samples (see Table 2). For the unamplified material, four patient samples (127, 146, 225, and 344) yielded anomalously high heterozygote:homozygote ratios (ranging from 4.5–4.9) and were therefore excluded from further comparative analyses. The transition:transversion ratio ranged from 2.0 to 3.4 for all samples. The average total number of variants detected in samples following WGA was 8073 for tumor tissue and 8277 for normal tissue (Table 2).

### Comparison of WGA and Unamplified Sequencing of Tumor DNA

**Figure 1:**
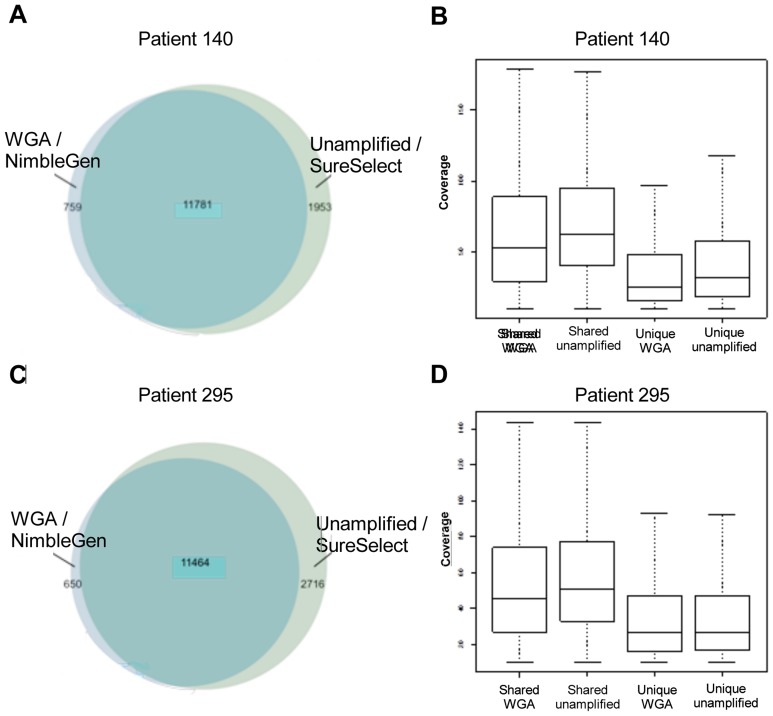
Genetic variants unique or shared between analysis. A) Venn diagram illustrating the distribution of SNVs for patient 140. Unique variants found by WGA are represented in light blue, and unique variants found without amplification are shown in light green. Shared variants identified by both methods are shown in green. B) Boxplot of the coverage of genetic variants found uniquely by WGA, without amplification, and with both methods for patient 140. The two leftmost boxes represent shared variant calls with coverage in those positions for WGA and without amplification, respectively. The two rightmost boxes represent the coverage over unique positions for each method. C) Venn diagram illustrating the distribution of SNVs for patient 295. Unique variants found by WGA are represented in light blue, and unique variants found without amplification are shown in light green. Shared variants identified by both methods are shown in green. D) Boxplot of the coverage of genetic variants found uniquely by WGA, without amplification, and with both methods for patient 295. The two leftmost boxes represent shared variant calls with coverage in those positions for WGA and without amplification, respectively. The two rightmost boxes represent the coverage over unique positions for each method.

**Figure 2:**
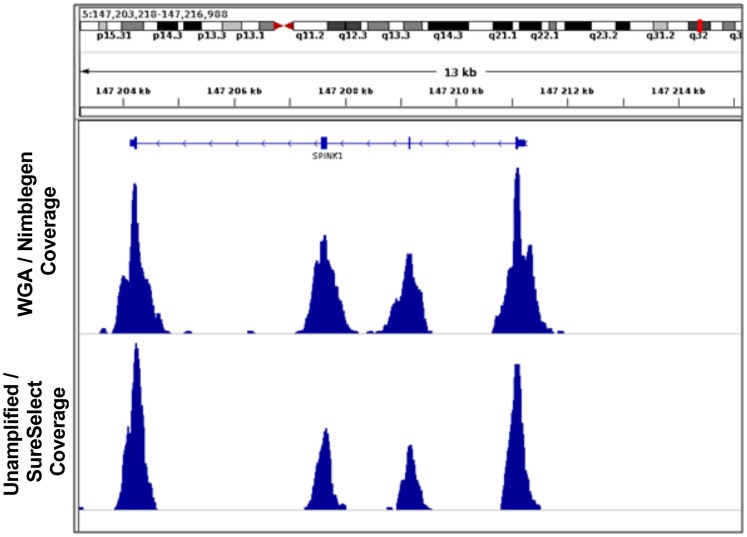
Example of coverage representation in unamplified and whole genome amplified DNA in patient 295 across the SPINK1 gene.

To identify biases that may be introduced by WGA, SNVs identified in the sequencing data obtained using WGA and unamplified material were compared using Venn diagrams (see Figure 1). The numbers of genetic variants called for WGA and unamplified material from tumor samples from two representative patients 140 and 295 are presented in Figures 1A and 1C. Most (74%) of the identified genetic variants were observed in both the amplified and the unamplified sequence data (Table 3). However, in most patients (83%) the number of unique variants identified in the unamplified material was greater than that observed following whole genome amplification. The coverage of genetic variants found uniquely by WGA and without amplification is presented using box plots for patients 140 and 295 in Figures 1B and 1D, along with coverage data for genetic variants observed in both sets of sequencing data (results for samples from the other patients considered are presented in Figure S1). The coverage of unique variants was consistently lower than that for common variants that could explain some of the discrepancies. Figure 2 provides a representative comparison of the results obtained with WGA and without amplification, showing the distribution of coverage over the exons of the gene SPINK1. Notably, the peaks for the WGA sequence are somewhat broader than those for the unamplified material in line with the overall lower coverage on target as compared to unamplified material for similar number of reads.

**Table 3:** Summary data for SNVs in positions identified in both WGA and unamplified sequence data, and using only one of the two methods.

Patient-ID	Number of SNV		
	WGA	Shared	Unamplified
118	722 (6%)	10985 (73%)	3260 (23%)
140	759 (6%)	11781 (81%)	1953 (14%)
210	1099 (9%)	11413 (75%)	2715 (19%)
247	1964 (17%)	9483 (59%)	4591 (33%)
255	606 (5%)	11162 (78%)	2615 (19%)
278	861 (7%)	12250 (84%)	1535 (11%)
295	650 (5%)	11464 (77%)	2716 (19%)
322	3866 (26%)	10821 (61%)	3023 (22%)
396	2076 (17%)	10151 (71%)	2119 (17%)
412	1559 (11%)	12216 (81%)	1374 (10%)
421	1204 (9%)	12108 (78%)	2133 (15%)
541	1398 (9%)	13350 (75%)	3096 (19%)
**Average (%)**	**1397 (9%)**	**11432 (74%)**	**2594 (17%)**
**StDev**	**923**	**1028**	**872**

**Figure 3:**
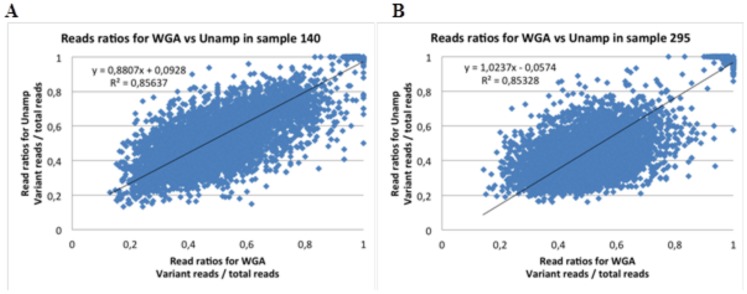
The relationship between variant and total reads per position depending on analysis. A) The relationship between the numbers of variant reads divided by total reads for SNVs identified by sequencing WGA and unamplified DNA per position in patient 140. B) The relationship between the numbers of variant reads divided by total reads for SNVs identified by sequencing WGA and unamplified DNA per position in patient 295.

To further evaluate the performance of the two methods in variant calling, we also compared the unamplified and WGA sequence data for patients 140 and 295 with respect to the number of variant reads divided by the total number of reads for each variant position (see Figure 3). Correlations for the remaining patients are presented in Figure S2 and Table S2. The ratios obtained using WGA were comparable to those for unamplified sequencing, with R^2^ values ranging from 0.79 to 0.88 in all cases except the four excluded samples.

Since on average only 74% of the total number of genetic variants found for each patient were observed in both the WGA and unamplified sequence data, we further examined the effect of coverage filtering on the discrepancies between genetic variants identified in WGA and unamplified material. This was done by calculating the fraction of variants that were only identified by WGA but were also present in the alignment files (BAM files without coverage filtering) for the unamplified sequences, and vice versa (Table 4). On average, 79% of the positions at which genetic variation was only observed in the sequence data for the unamplified material were identified in the aligned sequence BAM file for the WGA material with a coverage value of less than 10. For positions at which genetic variation was only observed in the WGA sequence data, only 36% were subsequently identified in the aligned sequence BAM files for the unamplified material with coverage values of less than 10, indicating a fraction of false positive variants in the WGA material.

**Table 4:** Comparison of unique and tumor specific variants in unamplified DNA, WGA DNA and RNA.

PatientID	% of uniqueunamplified withcoverage <10 inWGA bamfile	% of unique WGAwith coverage <10 inunamplified bamfile	% unique WGAin RNAwith coverage <10	WGA tumorspecific	% tumor specificconfirmed inunamplified	% WGA tumor specific inRNA (bam) withcoverage <10
118	71.7	57.6	69.9	1080	70.4	68.9
140	72.7	57.7	65.0	1644	77.9	64.2
210	84.8	21.8	62.5	630	33.3	59.4
247	90.9	5.7	–	2095	13.8	–
255	81.6	43.1	65.0	698	62.9	63.0
295	79.7	43.7	100.0	3510	81.5	100.0
278	73.3	50.9	63.4	1819	82.1	58.6
322	79.8	9.4	–	5377	11.4	–
396	79.7	53.6	64.9	1778	39.0	63.9
412	72.3	32.4	58.9	2580	60	60.5
421	78.3	27.2	100.0	994	38.1	100.0
541	82.0	25.9	–	1098	32.2	–
**Average**	**78.9**	**35.8**	**72.2**	**1942**	**50.2**	**70.9**
**StDev**	**5.7**	**18.1**	**16.0**	**1363**	**19.7**	**16.8**

### Identification of Tumor Specific Mutations

A key goal when sequencing tumor samples is to identify tumor specific mutations. We therefore compared the tumor-specific variants detected in the WGA sequence data to those for the unamplified material. On average, the WGA sequence data for the tumor samples contained 1942 tumor specific mutations (i.e. positions with coverage >10 for which no variation was detected in the WGA sequence data for the healthy tissue samples). To study these mutations more in detail, we compared the WGA results to those obtained for unamplified tumor sample DNA and were able to confirm the presence of just over half of them (50.2%; see Table 4).

### Validation of Identified Genetic Variants using RNA Sequencing

To determine whether it might be possible to use RNA-sequencing to validate mutation data, we calculated the fraction of tumor specific mutations (on average 1942) for which a corresponding position could be identified in the RNA sequence alignment files. On average, 71% of the mutations identified based on analysis of WGA sequence data were confirmed in this way (see the final column of Table 4).

To evaluate the use of RNA sequencing for variant calling, we used the number of bi-allelically expressed genes (i.e. genes with at least one heterozygous variant) and the list of the entire set of variants. We looked at the fraction of these genes for which analyses of the WGA and unamplified sequence data had also resulted in the identification of at least one genetic variant. In this comparison, we focused on transcripts with FPKM values above 2.8. To rule out mono-allelic expression, we chose transcripts for which two alleles were expressed (as indicated by the minor allele accounting for at least 20% of all transcripts) and compared the RNA sequence data to DNA sequence data for genes that exhibited variation when using either or both of the two tested sample preparation methods. Bi-allelic expression was observed for 37.6 percent of the expressed genes (representing the top 75% of all genes with FPKM>1). In total, 39% of the bi-allelic genes found by RNA sequencing also contained at least one genetic variant that was identified by sequencing of WGA and unamplified DNA. (See Table S2).

## Discussion

In general the coverage, total number of heterozygotes, homozygotes, transition/transversion rate and total number of reads were comparable for sequences produced by WGA and when using unamplified material, indicating that both methods worked well on most samples. Most (74%) of the SNVs identified in this work were observed in both the WGA sequence data and that for the unamplified material. Conversely, 9% of the identified SNVs were observed only in the WGA sequence data and 18% were observed only in the sequence data for the unamplified material. Of the SNVs found by both methods the read ratios for variant and total reads were also equivalent (R^2^ = 0.79–0.88, Table S1), but with some biased for the variant allele by WGA. The SNV that were identified by only one of the two sample preparation methods generally had lower coverage than those identified by both, indicating that these SNVs are located in regions that are more difficult to sequence. This is in accordance with the data presented by Indap *et al.* that also showed that down sampling bam files from sequencing of WGA samples results in reduction in accurately called variants [21]. When the search was expanded by removing the coverage filter, it was determined that 72% of the genetic variants found uniquely by sequencing unamplified material were present in the WGA alignment file. However a similar search expansion for the BAM file of the unamplified material identified only 36% of the genetic variants found in the filtered WGA data. This suggests that WGA might introduce some false positive genetic variation. Jiang *et al.* showed biased for WGA when comparing constitutional genetic variants called after WGA and shot-gun re-sequencing in a single sample, with an higher error rate for false negatives than false positives [22]. This difference compared to our results might be due to the presence of low frequency mutations in our material which might introduce a bias for the WGA.

All the WGA samples had satisfactory quality control results, including phred based quality scores, mapped reads values, and number of called variants, etc., and also had acceptable ratios of heterozygous/homozygous SNVs. However, four samples of the unamplified material did not satisfy this last criterion. Interestingly, of the four samples with higher heterozygous/homozygous ratios, sample 127 had a tumor content of 65%, which may have interfered with the relative abundance of heterozygotes and homozygotes. Samples 146 and 344 represented more morphologically aggressive cancers, with tumors at a later stage of development than was the case for the other samples. As a result, the sampled material may have been surrounded by tumor tissue with different levels of tumor progression. Since the unamplified method requires larger samples than are used with WGA, it is possible that they may have incorporated material with different levels of tumor development and would therefore exhibit a higher level of genetic variation than would be observed for the WGA samples. This highlights the importance of considering factors other than read depth and standard quality measures alone, even for samples that seem to satisfy conventional criteria. A more detailed analysis of these samples focusing on the read ratios and SNV coverage for the WGA and unamplified sequence data revealed an even more pronounced skew (see Figure S1 and S2). Also note that the difference in coverage and variants might also be due to the use of two different exome kits, however, we have tried to minimize the effects of this by only comparing the regions covered by both enrichment methodologies.

In cancer research, there is great interest in identifying tumor specific mutations [23], [24]. This was achieved by comparing the variants found in the WGA sequence data for tumor samples and healthy tissue, and excluding all variants present in the latter. On average, 1942 tumor-specific variants were identified in this way for each tumor sample, although only 46% of these could be confirmed by sequencing the unamplified tumor sample DNA. This should be compared to the observation that 89% of all SNVs (i.e. both tumor-specific and non-tumor specific SNVs, average 11432 shared variants) identified by sequencing tumor samples following WGA could be confirmed by sequencing unamplified material. This suggests that WGA introduces some false positive calls that are identified as mutations when the patient’s constitutional genetic variation is eliminated. Interestingly, 10% of the genetic variants found using both the unamplified and WGA protocols were mutations, i.e. variations identified by exome sequencing for both WGA and unamplified tumor DNA that were not present in the sequence of DNA from healthy samples. Of the SNVs found uniquely by WGA, 54% were identified as mutations. This value is comparable to the average number of confirmed mutations for this sample preparation method i.e. the fraction of WGA mutations (WGA pos and normal neg) found also by exome sequencing of unamplified tumor is almost the same as the fraction of mutations found only in the WGA exomes. Here we compared the coding genetic mutations found by standardized bioinformatic pipelines in WGA samples versus non-WGA samples. We have taken into account the coverage of the different capturing methods, and identified variants with and without a coverage filter. This has its limitation in that low coverage areas might be biased by inaccurate genotyping in these regions. However, in the coding region most bioinformatic pipelines do find mutations by comparing sequencing of both tumor and normal tissue. There are bioinformatic tools to deal with these issues like VarScan 2 [25] and pibase [26], but this might have the limitation of only studying high quality regions.

RNA-sequencing (RNA-seq) could potentially be useful for validating mutations identified in WGA sequence data. On average, we found that 71% of such mutations can be covered by RNA sequencing if the adequate sequencing depth can be achieved. However, the drawbacks of variant calling based on RNA sequence data are that it depends on bi-allelic expression and that there is a lack of variant calling tools that are designed for variant calling based on RNA-seq data. To eliminate the influence of bi-allelic expression on the variant calls, we determined the number of genes that were expressed bi-allelically (i.e. for which we found heterozygous SNVs) based on the RNA-seq data and also determined how many of these genes contained variants based on the WGA and unamplified tumor DNA sequence data. On average, 39% of the genes called with heterozygous variants in RNA-seq could also be verified by inspection of the WGA and unamplified tumor sequence data. This indicates that a large fraction of the variant calls obtained from RNA sequence data cannot be validated [27]. Although it is possible that the advent of rapid and affordable RNA sequencing might eliminate these discrepancies to some extent [28]. A further advantage of using RNA-seq data is that only genetic variants in highly expressed genes are identified.

Overall, the results presented herein indicate that the use of WGA in conjunction with sequence capture can identify a large fraction of the genetic variants found without using amplification when doing exome sequencing. However, a comparatively high fraction of the mutations identified using WGA alone cannot be confirmed using other methods. The main concern regarding the use of WGA in conjunction with sequence capture for mutation analysis is of course the introduction of false positives, which in this study was found to some extent. However, the use of WGA for identifying SNVs and constitutional genetic variants seems to be reliable, although caution should be taken when using WGA to identify mutations.

## Supporting Information

Figure S1 **Box-plots of the coverage of genetic variants found uniquely by WGA and without amplification as well as the coverage of shared genetic variants.** The two leftmost boxes represent shared variant calls with coverage in those positions for WGA and without amplification, respectively. The two rightmost boxes represent coverage over unique positions for each method.
(DOCX)

Figure S2 **The regression between the numbers of variant reads divided by total reads for SNV identified by sequencing of WGA and unamplified DNA per position.**
(DOCX)

Table S1 Summary of fitted regression line functions, with associated R^2^ correlation values, for all patients.
(DOCX)

Table S2 Fractions of bi-allelic genes for the unamplified and WGA sequence data.
(DOCX)
