# Supplementary material for: Assessment of Whole Genome Amplification for Sequence Capture and Massively Parallel Sequencing
Source: PLoS One. 2014 Jan 7;9(1):e84785. doi: 10.1371/journal.pone.0084785 (PMC3883664; doi:10.1371/journal.pone.0084785)
Supplement: Figure S1 — Box-plots of the coverage of genetic variants found uniquely by WGA and without amplification as well as the coverage of shared genetic variants. The two leftmost boxes represent shared variant calls with coverage in those positions for WGA and without amplification, respectively. The two rightmost boxes represent coverage over unique positions for each method. (DOCX) [file pone.0084785.s001.docx]

***Supplementary material***

***Supplemental Figure S1***

**Excluded samples**

******
