# Supplementary figures and images for: Assessment of Whole Genome Amplification for Sequence Capture and Massively Parallel Sequencing
Source: PLoS One. 2014 Jan 7;9(1):e84785. doi: 10.1371/journal.pone.0084785 (PMC3883664; doi:10.1371/journal.pone.0084785)

***Supplementary material***

***Supplemental Figure S2***


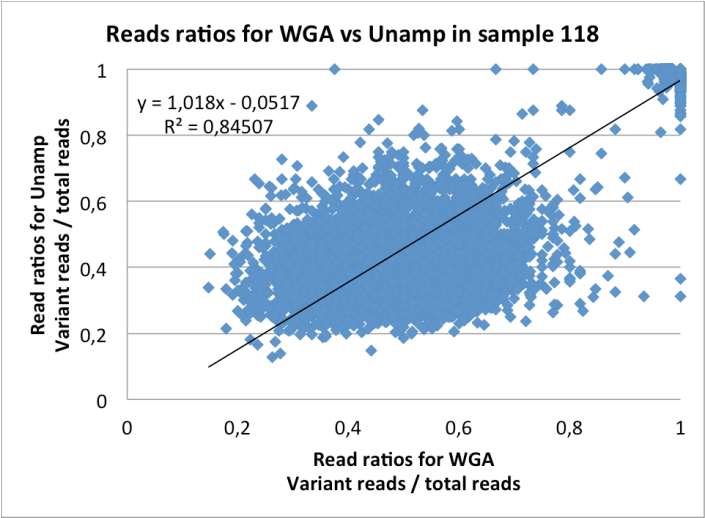

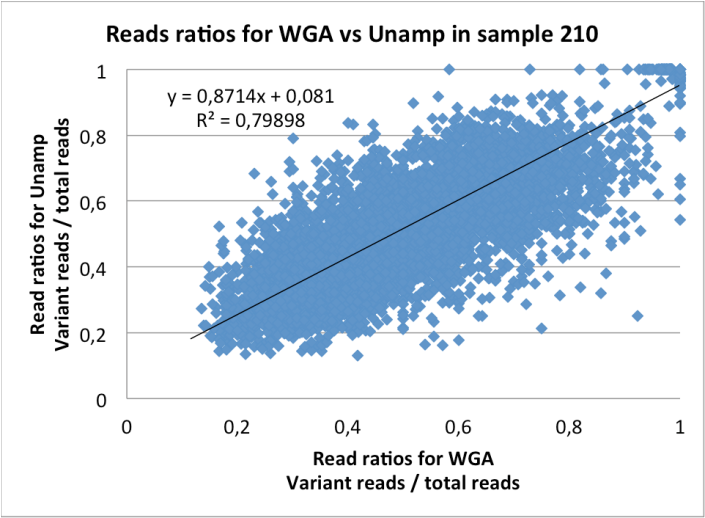


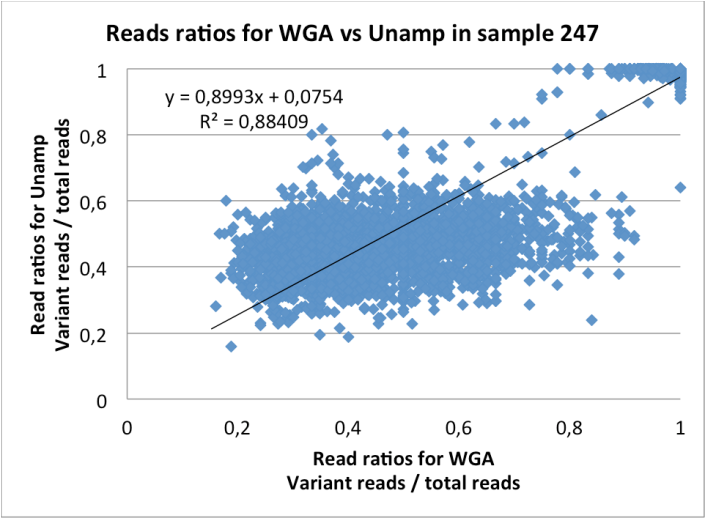

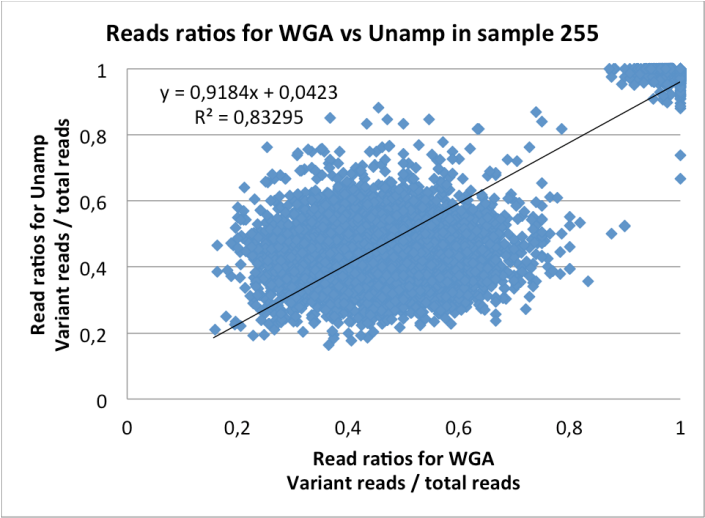


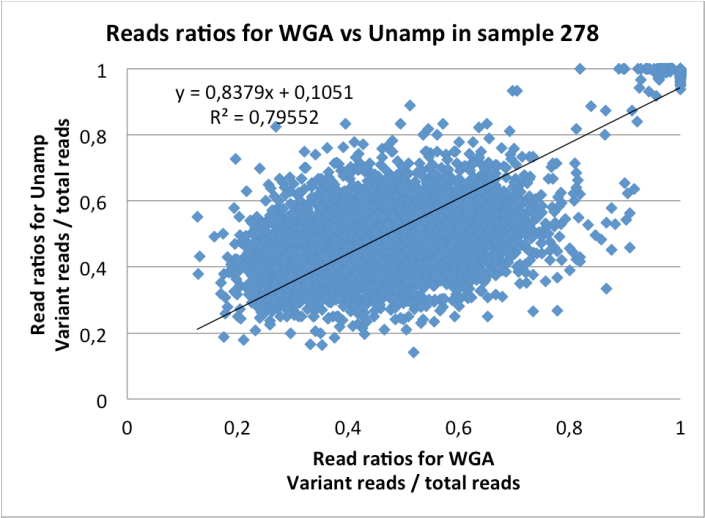

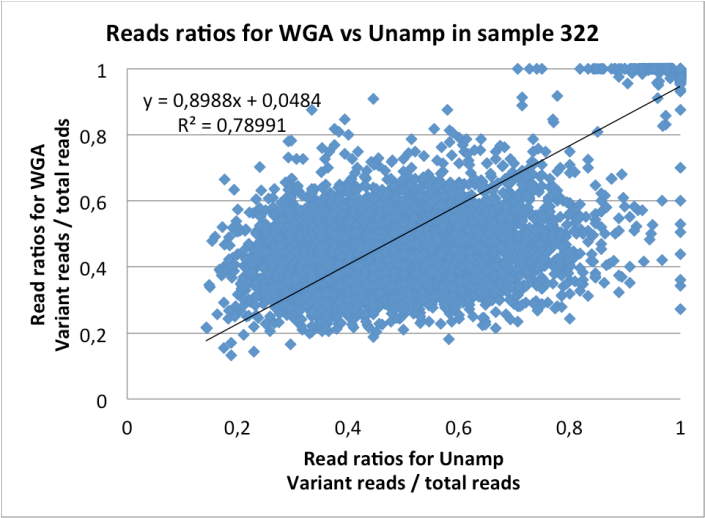


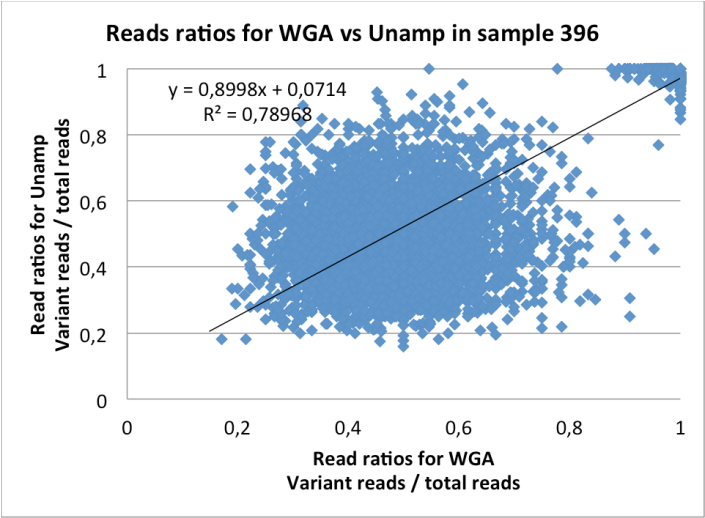

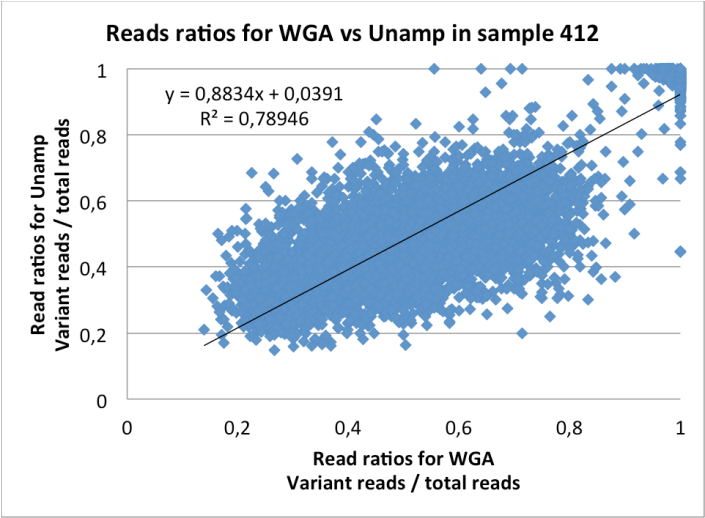


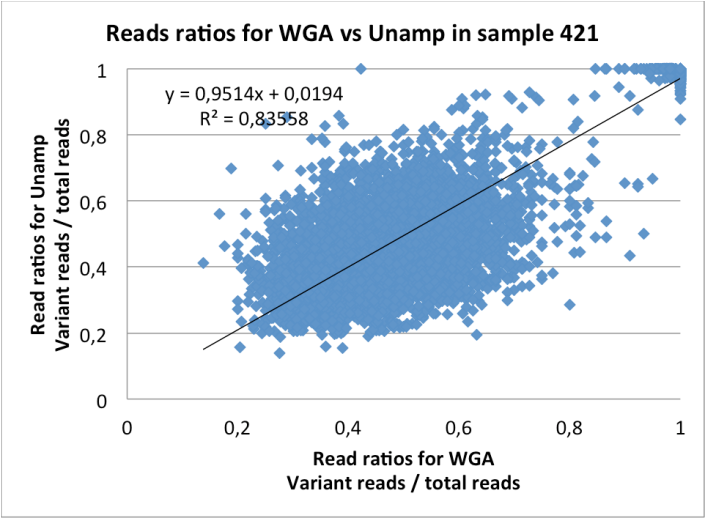

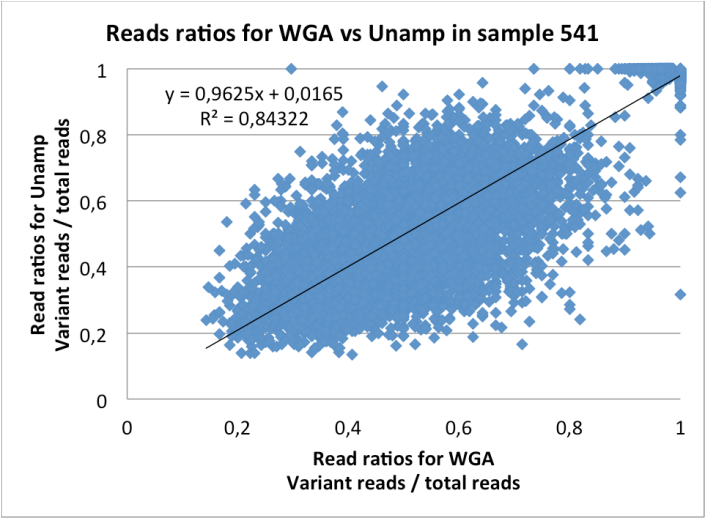


**Excluded samples**

**
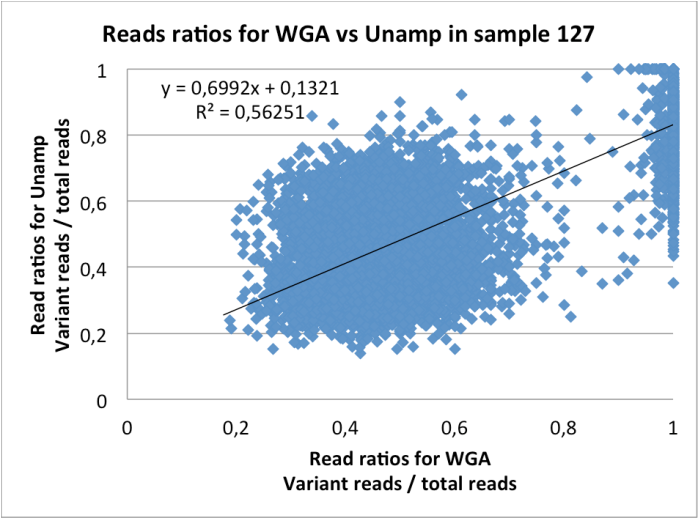

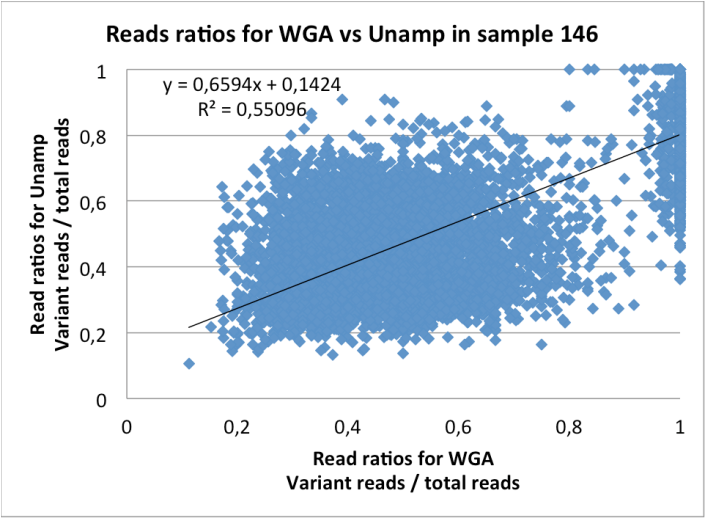
**

**
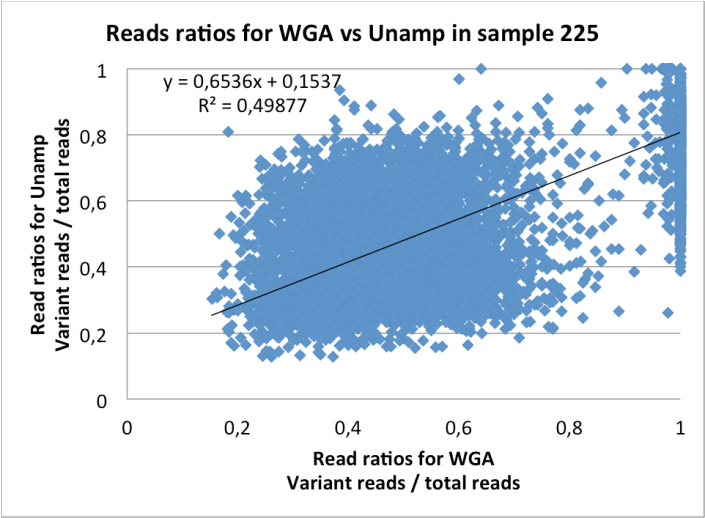

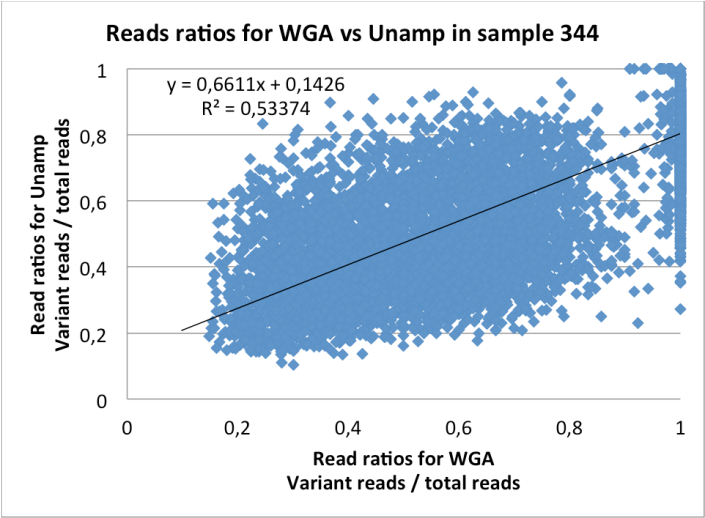
**

Supplement: Figure S2 — The regression between the numbers of variant reads divided by total reads for SNV identified by sequencing of WGA and unamplified DNA per position. (DOCX) [file pone.0084785.s002.docx]
