# Supplementary material for: Assessment of Whole Genome Amplification for Sequence Capture and Massively Parallel Sequencing
Source: PLoS One. 2014 Jan 7;9(1):e84785. doi: 10.1371/journal.pone.0084785 (PMC3883664; doi:10.1371/journal.pone.0084785)
Supplement: Table S1 — Summary of fitted regression line functions, with associated R2 correlation values, for all patients. (DOCX) [file pone.0084785.s003.docx]

***Supplementary material***

**Supplemental Table S1** – Summary of fitted regression line functions, with associated R^2^ correlation values, for all patients

| Patient ID | Regression line | R^2^ |
| --- | --- | --- |
| 118 | y = 1.02x - 0.05 | 0.85 |
| 140 | y = 0,88x + 0.09 | 0.86 |
| 210 | y = 0.87x + 0.08 | 0.80 |
| 247 | y = 0.90x + 0.08 | 0.88 |
| 255 | y = 0.92x + 0.04 | 0.83 |
| 278 | y = 0.84x + 0.11 | 0.80 |
| 295 | y = 1.02x - 0.06 | 0.85 |
| 322 | y = 0.90x + 0.05 | 0.79 |
| 396 | y = 0.90x + 0.07 | 0.79 |
| 412 | y = 0.88x + 0.04 | 0.79 |
| 421 | y = 0.95x + 0.02 | 0.84 |
| 541 | y = 0.96x + 0.02 | 0.84 |
