# Supplementary material for: Assessment of Whole Genome Amplification for Sequence Capture and Massively Parallel Sequencing
Source: PLoS One. 2014 Jan 7;9(1):e84785. doi: 10.1371/journal.pone.0084785 (PMC3883664; doi:10.1371/journal.pone.0084785)
Supplement: Table S2 — Fractions of bi-allelic genes for the unamplified and WGA sequence data. (DOCX) [file pone.0084785.s004.docx]

***Supplementary material***

**Supplemental Table S2** – Fractions of bi-allelic genes for the unamplified and WGA sequence data

| **Patient-ID** | **Bi-allelic expressed genes, total** | **Bi-allelic expressed genes, shared** | **%** |
| --- | --- | --- | --- |
| 118 | 2017 | 932 | 46.2 |
| 140 | 3802 | 1198 | 31.5 |
| 210 | 1810 | 875 | 48.3 |
| 255 | 2212 | 1046 | 47.3 |
| 278 | 4864 | 1245 | 25.6 |
| 295 | 2598 | 1108 | 42.6 |
| 396 | 4063 | 1205 | 29.7 |
| 412 | 2145 | 1027 | 47.9 |
| 421 | 3792 | 1289 | 34.0 |
| **Average** | **3034** | **1103** | **39.2** |
